# Lack of evidence of a relationship between magnetic resonance signal intensity changes in the globus pallidus and dentate nucleus, and repeated administrations of gadoterate meglumine in children

**DOI:** 10.1007/s00247-017-3947-1

**Published:** 2017-09-09

**Authors:** Eric Lancelot, Jean-Sébastien Raynaud, Pierre Desché

**Affiliations:** Guerbet BP57400, 95943 Roissy CDG Cedex, France

Dear Editors,

Rossi Espagnet and colleagues [1] reported significant increases of the globus pallidus-to-thalamus and the dentate nucleus-to-pons signal intensity ratios on unenhanced T1-weighted brain magnetic resonance images from children exposed to multiple injections of the macrocyclic gadolinium-based contrast agent gadoterate meglumine. However, this study has several important inconsistencies and limitations.

In the Materials and Methods section, the authors wrote that each control subject was matched to a patient for age at both the first and the last MR examinations. The groups did not differ statistically at baseline but the results of the comparison at the last examination were not presented, thus precluding any interpretation of the signal intensity ratio increases. Age-dependent changes in native T1-weighted MR contrast of the brain may well account for these effects.

In the Results section, the authors correlated the increases in the globus pallidus-to-thalamus and the dentate nucleus-to-pons signal intensity ratios to the number of gadolinium-based contrast agent injections. This association is not consistent with data from previous studies reporting an absence of correlation between these parameters in children after serial administrations of a linear gadolinium-based contrast agents [2, 3].

In Fig. 2, Rossi Espagnet and colleagues showed the relationships between the signal intensity ratios and the mean time intervals from the first administration. These graphs are misleading because the standard deviations of the time intervals at each injection were not presented. According to Table 1 in Rossi Espagnet's publication, the mean interval between MR examinations varied from 1 day to 532 days. It is likely that the mean time intervals reported in Fig. 2 were highly heterogeneous from one patient to another or between two injections in the same patient. Short time intervals of one to several days may have resulted in signal intensity increases due to incomplete wash-out of the gadolinium-containing molecules from the brain, and possibly to a significant signal intensity ratio increase in some patients.

The authors excluded the effect of a history of radiation therapy to the brain as a possible cause for the signal intensity ratio increases. However, it is probable that these patients with brain tumors underwent additional radiotherapy sessions during the study period. Blood–brain barrier disruption induced by radiotherapy may contribute to signal intensity increases following macrocyclic gadolinium-based contrast agent injection.

Some of the data reported by the authors in the Results section are not consistent with those that they presented graphically. The authors wrote that the globus pallidus-to-thalamus signal intensity mean value at first MR examination was 1.06±0.04 whereas it was rather equal to 1.048 according to Fig. 2. Such a mistake may have affected the statistical analyses.

Moreover, Flood and colleagues [3] found that the dentate nucleus-to-pons signal intensity ratio difference between the last and first MR examinations in children exposed to a linear gadolinium-based contrast agent was 1.035–0.995=0.04 [3]. In the present study, the difference was 1.02–0.95=0.07. It is difficult to understand how gadoterate meglumine could have triggered a greater signal intensity difference than a linear gadolinium-based contrast agent without inducing any visible enhancement.

Altogether, this exploratory study presented major inconsistencies that could have biased the interpretation of the results.
